# Rapid, Automated, and Specific Immunoassay to Directly Measure Matrix Metalloproteinase-9–Tissue Inhibitor of Metalloproteinase-1 Interactions in Human Plasma Using AlphaLISA Technology: A New Alternative to Classical ELISA

**DOI:** 10.3389/fimmu.2017.00853

**Published:** 2017-07-24

**Authors:** Helena Pulido-Olmo, Elena Rodríguez-Sánchez, José Alberto Navarro-García, María G. Barderas, Gloria Álvarez-Llamas, Julián Segura, Marisol Fernández-Alfonso, Luis M. Ruilope, Gema Ruiz-Hurtado

**Affiliations:** ^1^Laboratorio de Hipertensión y Riesgo Cardiovascular y Unidad de Hipertensión, Instituto de Investigación imas12, Hospital Universitario 12 de Octubre, Madrid, Spain; ^2^Facultad de Farmacia, Instituto Pluridisciplinar, Universidad Complutense de Madrid, Madrid, Spain; ^3^Laboratorio de Fisiopatologia Vascular, Hospital Nacional de Paraplejicos SESCAM, Toledo, Spain; ^4^Departamento de Inmunologia, IIS-Fundacion Jimenez Diaz, REDinREN, Universidad Autónoma de Madrid, Madrid, Spain; ^5^Departamento de Medicina Preventiva y Salud Pública, Universidad Autónoma de Madrid, Madrid, Spain; ^6^Escuela de Estudios Postdoctorales e Investigación, Universidad de Europa de Madrid, Madrid, Spain

**Keywords:** matrix metalloproteinase-9, tissue inhibitor of metalloproteinase-1, AlphaLISA^®^, protein interaction, immunoassay

## Abstract

The protocol describes a novel, rapid, and no-wash one-step immunoassay for highly sensitive and direct detection of the complexes between matrix metalloproteinases (MMPs) and their tissue inhibitor of metalloproteinases (TIMPs) based on AlphaLISA^®^ technology. We describe two procedures: (i) one approach is used to analyze MMP-9–TIMP-1 interactions using recombinant human MMP-9 with its corresponding recombinant human TIMP-1 inhibitor and (ii) the second approach is used to analyze native or endogenous MMP-9–TIMP-1 protein interactions in samples of human plasma. Evaluating native MMP-9–TIMP-1 complexes using this approach avoids the use of indirect calculations of the MMP-9/TIMP-1 ratio for which independent MMP-9 and TIMP-1 quantifications by two conventional ELISAs are needed. The MMP-9–TIMP-1 AlphaLISA^®^ assay is quick, highly simplified, and cost-effective and can be completed in less than 3 h. Moreover, the assay has great potential for use in basic and preclinical research as it allows direct determination of native MMP-9–TIMP-1 complexes in circulating blood as biofluid.

## Introduction

Matrix metalloproteinases (MMPs) belong to a large family of zinc-dependent endopeptidases that contribute to tissue remodeling by degrading extracellular matrix. MMPs are classified on the basis of their structure and substrate specificity into several groups: collagenases, gelatinases, stromelysins, matrilysins, and membrane type MMPs (1). MMP-9, also known as gelatinase B, is a type IV collagenase that can degrade denatured collagen (gelatin), elastin, and type IV collagen (2, 3). Alterations in the expression or activity of MMP-9 (and other MMPs) are associated with many pathological states related to inflammation including renal (1), cardiovascular (4, 5), autoimmune (6), degenerative (7), and neoplastic diseases (8), among others. MMP-9 activity is tightly regulated at three different levels, such as activation, synthesis, and inhibition: its synthesis is regulated by adhesion molecules such as integrins, cytokines, and growth factors (9); similar to other MMPs, MMP-9 is synthesized as a pro-enzyme and is activated by proteolytic cleavage of its pro-peptide domain by various proteases including plasmin, meprins, furins, and other activated MMPs (10); finally, it is inhibited by its endogenous inhibitor, tissue inhibitor of metalloproteinases 1 (TIMP-1), which inhibits active MMP-9 by interacting with its catalytic domain in a 1:1 (MMP-9:TIMP-1) stoichiometry (3). Accordingly, the MMP-9/TIMP-1 ratio is a commonly used metric to indirectly assess MMP-9 activity in experimental and preclinical settings (11–14). Solid-phase immunoassays such as the ELISA are the gold standard analytical platform used in preclinical laboratories for quantification of analytes in plasma, including MMP-9 and TIMP-1. While they provide high sensitivity and specificity, these conventional ELISA platforms used for estimating MMP-9–TIMP-1 protein interaction have several inherent limitations:

(i) they are labor intensive due to the multiple wash steps, making it difficult to adapt to high-throughput and automation;
(ii) the quantification of MMP-9 and TIMP-1 is very often carried out separately using two independent ELISA kits in preclinical studies, which doubles the analytical workload;
(iii) MMP-9/TIMP-1 ratio is not always a good surrogate measure for MMP-9–TIMP-1 interaction and much less an indirect indicator of MMP-9 activity. This is because traditional estimations of the MMP-9/TIMP-1 ratio do not consider that TIMP-1 can also be subjected to posttranslational modification under specific circumstances. For example, TIMP-1 can be oxidized which, as demonstrated recently (15, 16), modifies its inhibitory capacity on MMP-9. In this type of situations, calculating the ratio between total circulating MMP-9 and TIMP-1 as a surrogate measure of MMP-9–TIMP-1 interaction would be hiding that TIMP-1 is posttranslationally modified and thus could not interact with MMP-9 in order to inhibit it.

In light of these weaknesses, there is a clear need for a more simple and robust alternative for the precise and direct estimation of MMP-9 and TIMP-1 interactions in a high-throughput format with a stronger application in basic and preclinical research. Bead-based AlphaLISA^®^ immunoassays have been designed for the detection of analytes in biological samples (17). AlphaLISA^®^ immunoassay (amplified luminescent proximity homogeneous assay, ALPHA) is a bead-based non-radioactive assay that uses luminescent oxygen-channeling chemistry (17, 18). We have recently developed a specific AlphaLISA^®^-based assay to directly analyze MMP-9–TIMP-1 complexes in human plasma (16). Here, we describe the step-by-step protocol of this no-wash, rapid, and one-step homogeneous immunoassay for automatically determining specific MMP-9–TIMP-1 complexes using both recombinant human MMP-9 and TIMP-1 proteins and also endogenous/native MMP-9 and TIMP-1 proteins from samples of human plasma.

## Materials and Equipment

### Chemicals/Reagents

– HEPES ≥99.5% (Cat. No. H7523, Sigma-Aldrich).
– Sodium cyanoborohydride (NaBH_3_CN) (Cat. No. 156159, Sigma-Aldrich)! **CAUTION** Sodium cyanoborohydride is flammable, corrosive, and acutely toxic (by oral, dermal, and inhalation exposure). Wear protective clothing, eyewear, and gloves when handling this reagent. Sodium cyanoborohydride is also hazardous to the aquatic environment.
– Carboxymethoxylamine (CMO) (Cat. No. C13408, Sigma-Aldrich).
– Tris (Cat. No. 161-0719, BioRad)! **CAUTION** Acutely toxic (by oral, dermal, and inhalation exposure). Wear protective clothing, eyewear, and gloves when handling.
– NaOH (Cat. No. S5881, Sigma-Aldrich,)! **CAUTION** Corrosive, avoid direct contact with NaOH, and wear protective clothing when handling this reagent.
– HCl (Cat. No. 211019, Química Moncada)! **CAUTION** Corrosive, avoid direct contact with HCl, and wear protective clothing when handling this reagent.
– 10% Tween 20 (Cat. No. 28320, Thermo Fisher Scientific Inc.).
– Proclin-300 (Cat. No. 48912-U, Sigma-Aldrich)! **CAUTION** Corrosive and acutely toxic (by oral, dermal, and inhalation exposure). Wear protective clothing, eyewear, and gloves when handling this reagent.
– NaCl (Cat. No. S7653, Sigma-Aldrich).
– BSA (Cat. No. A7030, Sigma-Aldrich).
– PBS (Cat. No. 10010-023, Invitrogen Life Technologies).
– Anti-MMP-9 antibody (Cat. No. MS817PABX, Thermo Fisher Scientific).
– Biotinylated anti-TIMP-1 antibody (Cat. No. MA5-13685, Thermo Fisher Scientific).
– AlphaLISA^®^ Acceptor beads 1 mg (Cat. No. 6772001, PerkinElmer).
– AlphaLISA^®^ Streptavidin Donor beads 1 mg (Cat. No. 6760002S, PerkinElmer).
– Recombinant TIMP-1 (rTIMP-1) human (Cat. No. SRP3173, Sigma-Aldrich).
– Recombinant MMP-9 (rMMP-9) human (Cat. No. 10327-H08H-50, Invitrogen Life Technologies).
– Human plasma for testing endogenous MMP-9–TIMP-1 complexes. In our study, a total of 36 patients aged >18 years with primary hypertension were recruited from the Hypertension Unit at Hospital Universitario 12 de Octubre, Madrid. Exclusion criteria included patients with diabetes mellitus or primary hyperaldosteronism. Patients were classified as normoalbuminuria when albumin/creatinine ratio was maintained <20 mg/g for men and <30 mg/g for women, and as albuminuria for those patients who reported abnormal urinary albumin excretion that remained above target levels (albumin/creatinine ratio ≥20 mg/g for men and ≥30 mg/g for women). The study was approved by the Ethics Committee of the Hospital Universitario 12 de Octubre and was conducted according to the principles of the Declaration of Helsinki. All patients signed a written informed consent before inclusion.

### Equipment

– Microtube 1.5 mL (Cat. No. 72690001, Sarstedt).
– Tube 15 mL (Cat. No. 62554502, Sarstedt).
– Tube 50 mL (Cat. no. 210261, Greiner bio-one).
– Micropipettes (Rainin, L-10XLS+, L-20XLS+, L-200XLS+, and L-1000XLS+).
– Pipette tips [Cat. No. 163031 (0.2–10 µL); 162001 (5–200 µL); 162222 (100–1,000 µL), Daslab].
– Bench centrifuge (MiniSpin Plus, Eppendorf).
– Sonicator (Model F60, Fisher).
– Rotary shaker (Cat. No. 88880028, Thermo Scientific).
– Vortex Mixer (Cat. No. F202A0173, Velp Scientifica).
– pH meter (Cat. No. FP20, Mettler Toledo).
– Mili-Q System (Millipore).
– Assay Plate 96-well untreated (Cat. No. 6005560, PerkinElmer).
– EnSpire™ Multimode Plate Reader (version 4.1; Cat. No. 2300, PerkinElmer). See Equipment Setup.

### Reagents Setup

– *800 mM NaOH*: dissolve 3.2 g of NaOH in 100 mL of Milli-Q water.
– *100 mM HEPES, pH 7.4*: dissolve 2.38 g of HEPES in 100 mL of Milli-Q water. Once completely dissolved, adjust pH carefully with HCl.
– *100 mM Tris–HCl, pH 8.0*: dissolve 1.21 g of Tris in 100 mL of Milli-Q water. Once completely dissolved, adjust pH carefully with HCl.
– *Storage buffer, 0.05% Proclin-300*: dilute 2 µL of Proclin-300 in 3,998 µL of 1× PBS
– *Fresh working solution 400 mM NaBH_3_CN*: dissolve 25 mg of NaBH_3_CN in 1 mL of Milli-Q water.! **CRITICAL** Do not store, prepare immediately before use.
– *Fresh working CMO solution*: dissolve 65 mg of CMO in 800 mM of NaOH.! **CRITICAL** Do not store, prepare immediately before use.
– *Assay buffer*: dissolve 6 mg of HEPES and 584 mg of NaCl in 80 mL of Milli-Q water. Adjust pH to 7.4 with 800 mM NaOH. Then carefully dissolve 100 mg of BSA, avoiding foaming of the solution. Finally, add 50 µL of 10% Tween-20 and adjust with of Milli-Q water to obtain a final volume of 100 mL.
– *5 nM biotinylated anti-TIMP-1 antibody*: a predilution of 100 nM TIMP-1 antibody is required. To obtain a final volume of 50 µL, add 4.2 µL of the stock solution to 45.8 µL of assay buffer in a 1.5-mL microtube. Immediately add 399 µL of assay buffer into a new 1.5 mL microtube and add 21 µL of the prediluted TIMP-1 antibody to obtain a final volume of 420 µL.
– *Anti-MMP-9 AlphaLISA^®^Acceptor Beads mix*: dilute the AlphaLISA acceptor beads stock solution 100-fold in AlphaLISA assay buffer to obtain a final concentration of 10 µg/mL. The stock solution can be stored at 4°C up to 6 months.
– *AlphaLISA^®^Streptavidin Donor Beads mix*: dilute the AlphaLISA^®^ donor beads stock solution 50-fold in AlphaLISA^®^ assay buffer to obtain a final concentration of 40 µg/mL.! **CRITICAL** The donor beads are light sensitive, so work must be performed in subdued light. The stock solution can be stored at 4°C up to 6 months.
– *For recombinant human MMP-9 and TIMP-1 immunoassay*:
  ○ *TIMP-1, 1 mg/mL stock solution*: reconstitute the lyophilized protein of 10 µg in 10 µL of Milli-Q water to obtain a final concentration of 1 mg/mL.
  ○ *TIMP-1, 0.003 mg/mL intermediate solution*: put 3 µL of 1 mg/mL Stock solution in 997 µL of Milli-Q water to obtain a final concentration of 0.003 mg/mL.
  ○ *MMP-9, 0.1 mg/mL stock solution*: reconstitute the lyophilized protein of 5 µg in 50 µL of Milli-Q water to obtain a final concentration of 0.1 mg/mL.
  ○ Working solutions are prepared as shown in Table 1. The final concentration in the immunoassay will be 0, 30, 100, 300, and 1,000 ng/mL.
  ○ Blank luminescence signal corresponding to 0 ng/mL of MMP-9 and 0 ng/mL of TIMP-1 is subtracted from all reading points.
– *For endogenous human plasma MMP-9 and TIMP-1 assay*: 5 µL of human plasma. Blood samples are collected in tubes containing heparin lithium and centrifuged at 900 × *g* for 4°C to obtain plasma. Samples can be frozen at −80°C.! **CAUTION** Plasma samples can be stored at −80°C up to 12 months.

**Table 1:** Troubleshooting guide.

Step	Problem	Possible reason	Solution
3, 15, 18	Low number of acceptor or donor beads in wells	Poor bead suspension	Mix the stock bead suspension gently, avoid foaming when vortexing bead solutions before use

13	Deteriorated stored AlphaLISA^®^ acceptor beads	Storage of AlphaLISA^®^ acceptor beads longer than 6 months or incorrectly stored	Conjugated acceptor beads must be properly stored at 4°C in an opaque vial, carefully closed to avoid degradation or evaporation. Do not store conjugated acceptor beads longer than 6 months

14, 17, 18	Lower signal than expected	Low incubation temperature	Component binding is optimal at 20–25°C, check lab temperature to ensure that it is within this range
		Short incubation time	Ensure correct incubation time
	High degree of signal variability	Uneven well evaporation	Use a top cover plate during incubations
		Air bubbles in some wells	Use electronic pipettes, avoid foam when vortexing bead solutions before use

14A	Hook effect	The bead system (donor and acceptor beads) have become to be saturated when the maximum binding capacity is reached and an excess of matrix metalloproteinase (MMP)-9 or tissue inhibitor of metalloproteinase (TIMP)-1 is able to dissociate the interaction between the two beads provoking a decreased RLU signal	The hook point in our assay is reached at 300 ng/mL MMP-9 and TIMP-1. Since the hook effect is a common intrinsic phenomenon present in any no-wash sandwich-type assay as this, we recommend to use MMP-9 and TIMP-1 concentration below this hook point

14 B	Signal intensity is very high	Concentration of MMP-9–TIMP-1 complexes of the sample is too high	Dilute samples
	No signal, lower signal than expected or high background signal	Contamination or deterioration of assay buffer	Prepare fresh assay buffer before use

14, 15, 16, 18	High degree of signal variability	Mixing problems	Gently tap the plate against a hard surface to ensure all reagents are at the bottom of the well

18	No signal or lower signal than expected	Donor beads have been exposed to light	Use non-light exposed beads. Always protect beads from light
	High background signal	Donor beads have been exposed to light	During incubation, use a black top cover or place the plate in a drawer

### Equipment Setup

#### 2300 EnSpire™ Reader

Use the plate reader with the following AlphaLISA^®^ standard protocol settings: excitation filter wavelength at 680 nm, emission filter wavelength at 615 nm, measurement time 200 ms, excitation time 80 ms (40%), and the distance between plate and detector 0.2 mm.

## Stepwise Procedures

### Antibody Conjugation to AlphaLISA^®^ Acceptor Beads

**Figure 1:**
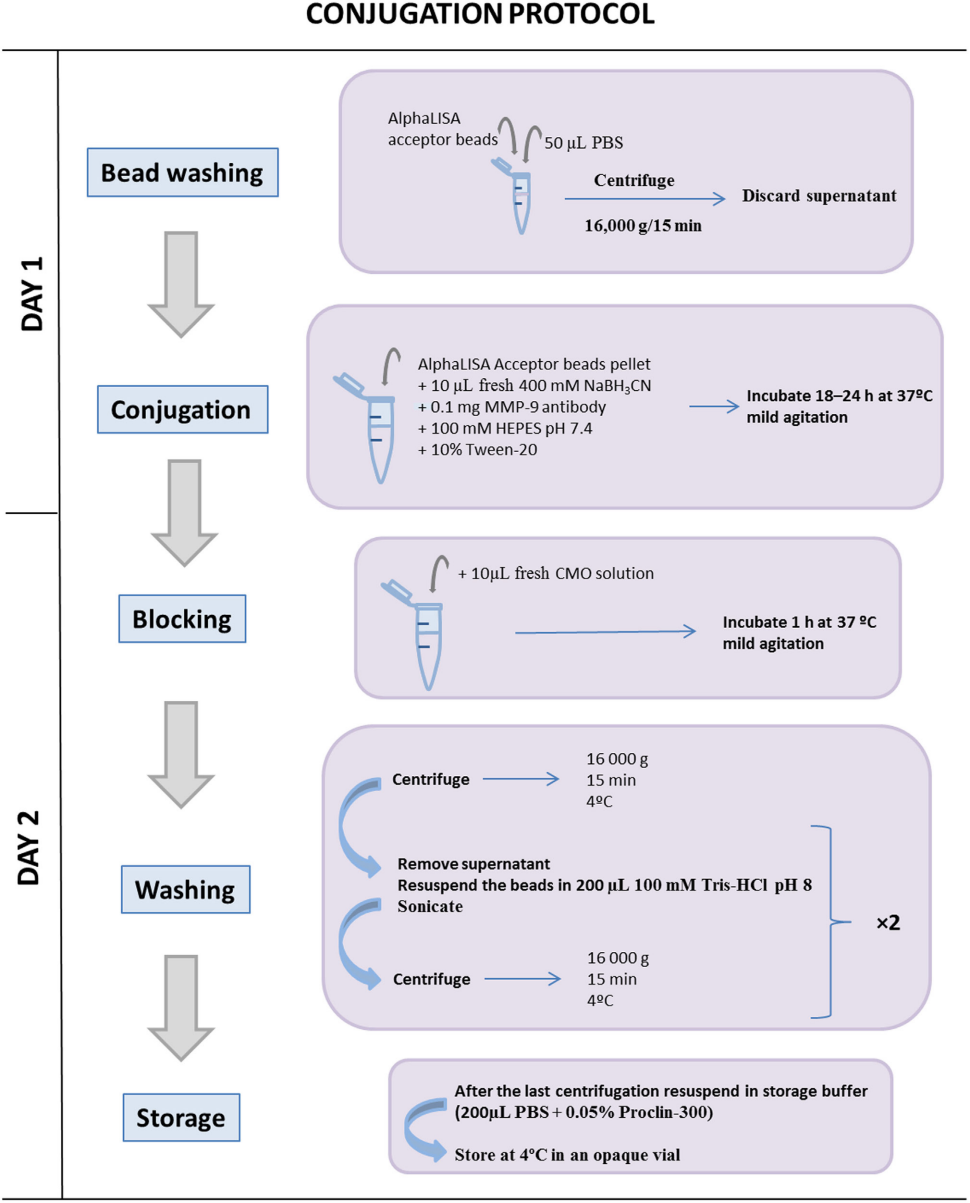
Schematic illustration of the conjugation protocol between the AlphaLISA^®^ acceptor bead and the matrix metalloproteinase (MMP)-9 antibody. Once the acceptor beads and the MMP-9 antibody are coupled, it is important to block free acceptor bead-binding sites to avoid further interactions with other antibodies. One conjugation between 1 mg AlphaLISA^®^ acceptor beads and 0.1 mg MMP-9 antibody reaches for a total 2,000 different interaction assays during 12 months.

AlphaLISA^®^ acceptor beads conjugation with anti-MMP-9 antibody in 10:1 coupling ratio (see Figure 1).

### Bead Washing Step TIMING 30 min

1. Transfer AlphaLISA^®^ acceptor beads to a 1.5-mL microtube.
2. Add 50 µL of PBS and pulse vortex to mix and centrifuge at 16,000 × *g* for 15 min at room temperature and then carefully remove the supernatant using a pipette.
  **CRITICAL STEP** To prevent bead loss, do not invert the tube.

### Conjugation Step TIMING 18 h

3. Add to the microtube containing 1 mg of AlphaLISA^®^ acceptor beads, plus 0.1 mg of the MMP-9 antibody, plus the appropriate volume of 100 mM HEPES pH 7.4 and pulse vortex to mix to obtain a final reaction volume of 200 µL; then add 1.25 µL of 10% Tween-20 and add 10 µL of 400 mM fresh NaBH_3_CN working solution and pulse vortex to mix. **CRITICAL STEP** This solution must be prepared freshly immediately before step 3. Discard any leftover solution.
  **? TROUBLESHOOTING** (see Table 1)
4. Incubate the microtube for 18 h at 37°C with mild agitation (6–10 rpm) in a rotary shaker.

### Blocking Step TIMING 1 h

5. Add 10 µL of CMO solution to the microtube and pulse vortex to mix to block unconjugated sites. **CRITICAL STEP** This solution must be prepared before use. Discard any leftover solution.
6. Incubate the microtube for 1 h at 37°C with mild agitation (6–10 rpm) using a rotary shaker.

### Washing Step TIMING 1 h

7. Centrifuge the microtube at 16,000 × *g* for 15 min at 4°C.
8. Remove the supernatant using a micropipette and carefully resuspend the pellet in 200 µL of 100 mM Tris–HCl pH 8.0.
9. Sonicate the bead solution for 10 pulses of 1 s. Adjust the sonicator power so not to exceed 20% of its maximal power. **CRITICAL STEP** A probe sonicator should be used rather than a bath sonicator. Sonication must be performed on ice, avoiding prolonged sonication which can lead to sample heating.
10. Repeat steps 7, 8, and 9.
11. After the final centrifugation step, resuspend the beads in 200 µL of storage solution to obtain a final bead concentration of 5 mg/mL.
12. Vortex and sonicate the solution using the same conditions as in step 9.
13. The conjugated acceptor beads stock solution should be stored at 4°C in an opaque vial.
  **? TROUBLESHOOTING** (see Table 1)

One conjugation between 1 mg AlphaLISA^®^ acceptor beads and 0.1 mg MMP-9 antibody reaches for a total 2,000 different interaction assays during 12 moths.

### Interaction Assay

**Figure 2:**
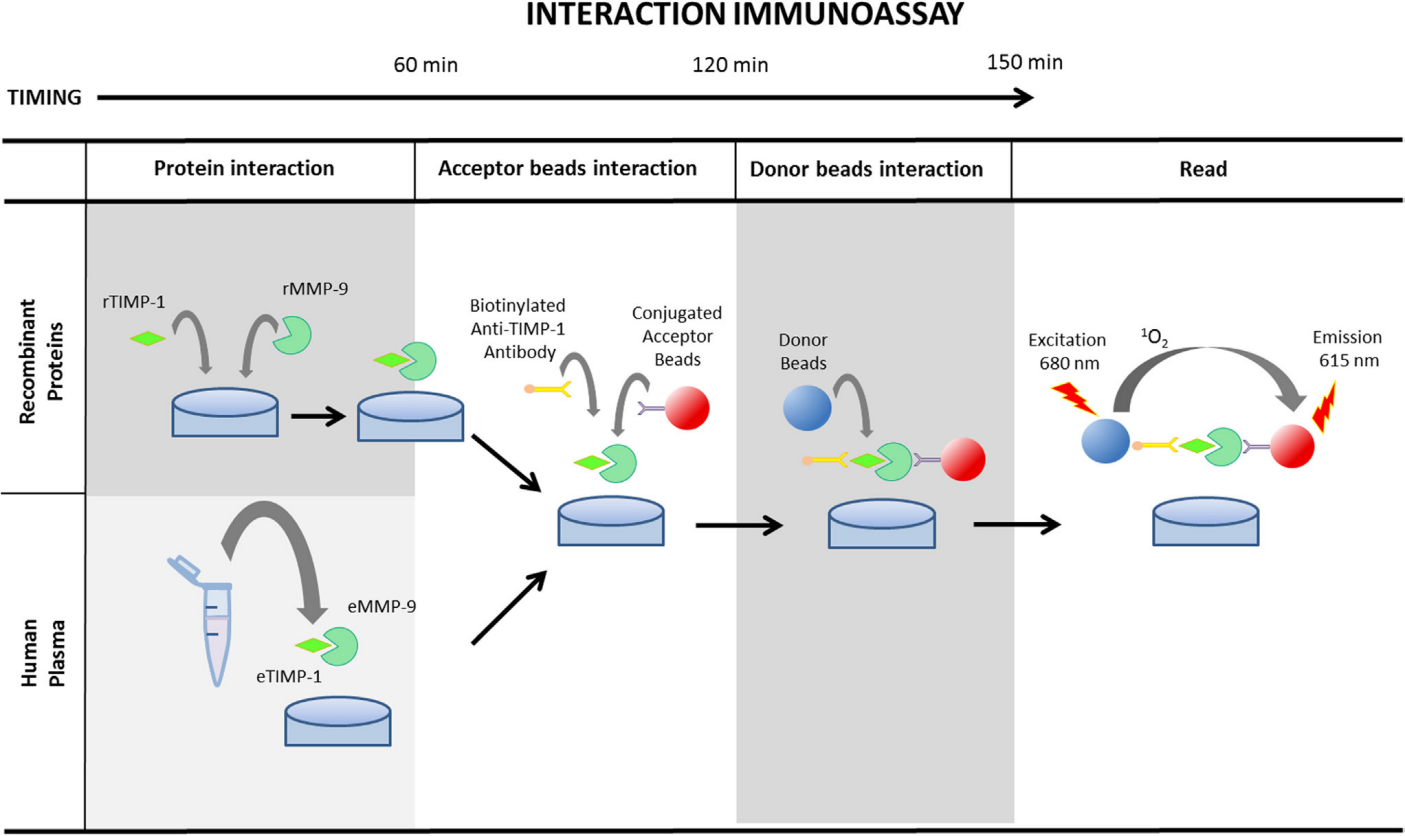
Schematic illustration of AlphaLISA^®^ protein interaction determination protocol using recombinant matrix metalloproteinase (MMP)-9 and tissue inhibitor of metalloproteinase (TIMP)-1 proteins or endogenous MMP-9 and TIMP-1 in human plasma. MMP-9 and TIMP-1 recombinant proteins need 1 h incubation at room temperature to promote the interaction. The subsequent step consists of the incubation of the sample or the recombinant proteins with the conjugated acceptor beads and the biotinylated anti-TIMP-1 antibody for an additional 1 h. Finally, after the incubation of the mixture with the donor beads for 30 min, the plaque should be read at 680 nm.

TIMING 3 h 40 min for 14A (recombinant human MMP-9 and TIMP-1 proteins) and 2 h 20 min for 14B (endogenous human plasma MMP-9 and TIMP-1 proteins) (see Figure 2). Final volume per well should be always 50 µL.

14. Add samples to the half-area of a 96-well plate
  First option 14A: human rMMP-9 and TIMP-1 proteins:
  (i) Add 5 µL of each recombinant human MMP-9 and 5 µL of each recombinant human TIMP-1 working solutions (points: 0, 300, 1,000, 3,000, and 10,000 ng/mL; see Table 2) (see Reagents Setup; dilution factor is 10, and for this the final concentration of the curve is 0, 30, 100, 300, and 1,000 ng/mL).
  (ii) Incubate plate for 60 min at room temperature.
  **? TROUBLESHOOTING** (see Table 1)
  OR
  Second option 14 B: human plasma samples: add 5 µL of plasma sample and 5 µL of assay buffer to the same well.
  **! CAUTION** Wear suitable protection (such as gloves and laboratory coat) when handling human samples.
  **? TROUBLESHOOTING** (see Table 1)
15. Add 10 µL of anti-MMP-9 AlphaLISA^®^ acceptor beads mix to each well to obtain a final concentration of 10 µg/mL (see Reagents Setup).
  **? TROUBLESHOOTING** (see Table 1)
16. Add 10 µL of 5 nM biotinylated anti-TIMP-1 to obtain a final concentration of 1 nM.
  **CRITICAL STEP** The use of a biotinylated antibody is mandatory to facilitate the interaction between the antibody and the donor bead.
17. Incubate the plate for 60 min at room temperature.
  **? TROUBLESHOOTING** (see Table 1)
18. Add 20 µL of AlphaLISA^®^ Streptavidin Donor Beads mix to obtain a final concentration of 40 µg/mL and incubate the plate for 30 min at room temperature (see Reagents Setup).
  **CRITICAL STEP** Any manipulation of AlphaLISA^®^ donor beads must be done in subdued light as they are light sensitive.
  **? TROUBLESHOOTING** (see Table 1)
19. Read the plate on 2300 EnSpire™ Reader

**Table 2:** Overview of how to prepare working solutions of recombinant matrix metalloproteinase (MMP)-9 and tissue inhibitor of metalloproteinase (TIMP)-1 for AlphaLISA^®^ immunoassay interaction.

Working solutions (ng/mL)	Recombinant TIMP-1 (rTIMP-1)		Recombinant MMP-9 (rMMP-9)	
	rTIMP-1 (μL)	Buffer (μL)	rMMP-9 (μL)	Buffer (μL)
10,000	0.4 µL of 1 mg/mL stock solution	39.6	4 µL of 0.1 mg/mL stock solution	36
3,000	40 µL of 0.003 mg/mL intermediate stock solution	0	1.2 µL of 0.1 mg/mL stock solution	38.8
1,000	13.3 µL of 0.003 mg/mL intermediate solution	26.7	4 µL of 10,000 ng/mL working solution	36
300	4 µL of 0.003 mg/mL intermediate solution	36	4 µL of 3,000 ng/mL of working solution	36
0	0 µL	40	0 μL	40

### Statistical Analysis

20. Statistical analysis is performed using Student’s *t*-test with GrapPad Prism 6.0. Data are reported as mean ± SEM Set the significance to a threshold of *P* < 0.05, and *n*-values represent the number of replicates or patients.

## Timing

Steps 1–3: Bead washing and conjugation: 30 min

Step 4: Conjugation incubation: 18 h

Steps 5–6: Blocking of unconjugated site: 1 h

Steps 7–13: Washing conjugated acceptor beads: 1 h

Step 14A: Addition of recombinant proteins into the plate and interaction: 1.5 h

OR

Step 14B: Addition of human plasma samples: 10 min

Steps 15–17: Addition of biotinylated anti-TIMP-1 antibody and conjugated acceptor beads and incubation: 1.5 h

Steps 18–19: Addition of donor beads and incubation: 40 min

**? TROUBLESHOOTING**

Troubleshooting advice can be found in Table 1.

## Anticipated Results

The activity of MMP-9 is controlled by TIMP-1, and both members of the MMP-9–TIMP-1 tandem are implicated in several disease states including cardiovascular and renal disease. Thus, alterations in plasma MMP-9 and TIMP-1 levels are related to a pathological remodeling of target organs as heart, vessels, or kidney during the development of diabetic nephropathy, albuminuria, arterial stiffness, or hypertension (16, 19–21), among other pathological situations. For that reason, an adequate, accurate, and direct measurement of the MMP-9–TIMP-1 interactions is completely necessary and has broad preclinical applicability since it would avoid indirect estimations derived from MMP-9/TIMP-1 calculations using classical ELISAs which may lead to unreliable results. One example of this situation occurs when posttranslational modifications of TIMP-1, as for example oxidation (15), impair TIMP-1 function through the loss of its inhibitory capacity on MMP-9, allowing a pathological and maintained activation of this metalloproteinase.

**Figure 3:**
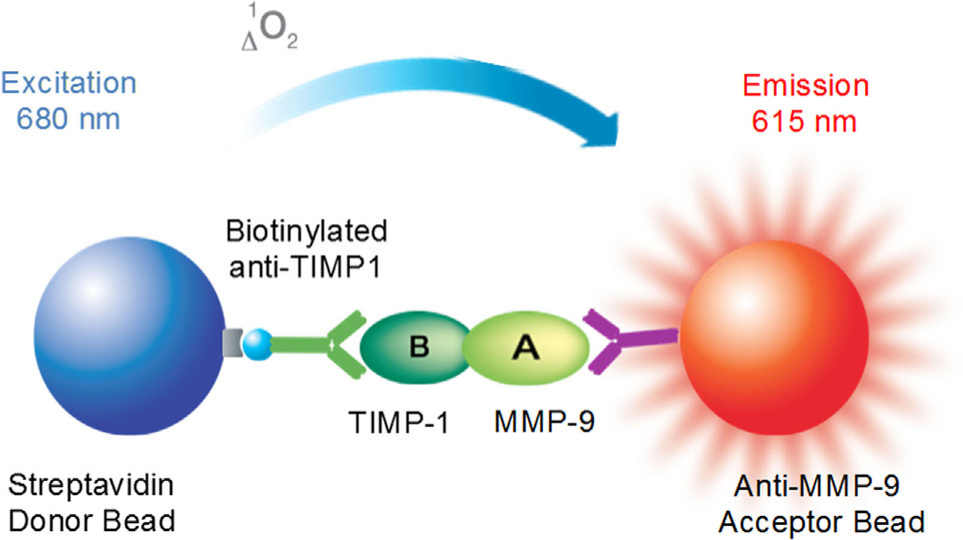
Principle of the AlphaLISA^®^ matrix metalloproteinase (MMP)-9–tissue inhibitor of metalloproteinase (TIMP)-1 interaction immunoassay.

Here, we utilized the AlphaLISA^®^ platform as a no-wash sandwich immunoassay to directly evaluate MMP-9–TIMP-1 interactions. The principle of the assay and the detection technology is illustrated by the scheme shown in Figure 3. The assay detects the proximity of donor and acceptor beads conjugated to biological “binding” partners, which leads to an energy transfer from one bead to the other. Thus, MMP-9 is captured on the acceptor bead, which is conjugated with the anti-MMP-9 antibody, whereas TIMP-1 is captured on a biotinylated anti-TIMP-1 antibody directly coupled to the donor bead. The donor bead is coated with streptavidin, which presents high affinity for biotin, and also with phthalocyanine, a photosensitizer that converts ambient oxygen to singlet oxygen when excited at 680 nm. When the acceptor bead is within 200 nm of the donor bead, by a molecular binding event between MMP-9 and TIMP-1, the singlet oxygen generated excites thioxene derivatives within the acceptor bead, resulting in light production at 615 nm. The light emitted is proportional to the level of MMP-9–TIMP-1 complexes contained in the sample. In the absence of biological interaction between MMP-9 and TIMP-1, the singlet state oxygen molecules produced by the donor beads is undetected. It is important to note that the choice of AlphaLISA^®^ technology over AlphaScreen^®^ was taken to avoid the overlap of the emission wavelength between AlphaScreen^®^ and heme groups contained in plasma samples. Here, we used the MMP-9–TIMP-1 AlphaLISA^®^ immunoassay to detect: (i) MMP-9–TIMP-1 immunocomplexes using recombinant human MMP-9 and TIMP-1 proteins; (ii) endogenous/native MMP-9–TIMP-1 immunocomplexes directly in human plasma samples.

### AlphaLISA^®^ Immunoassay to Detect MMP-9 and TIMP-1 Interactions Using Recombinant Proteins

**Figure 4:**
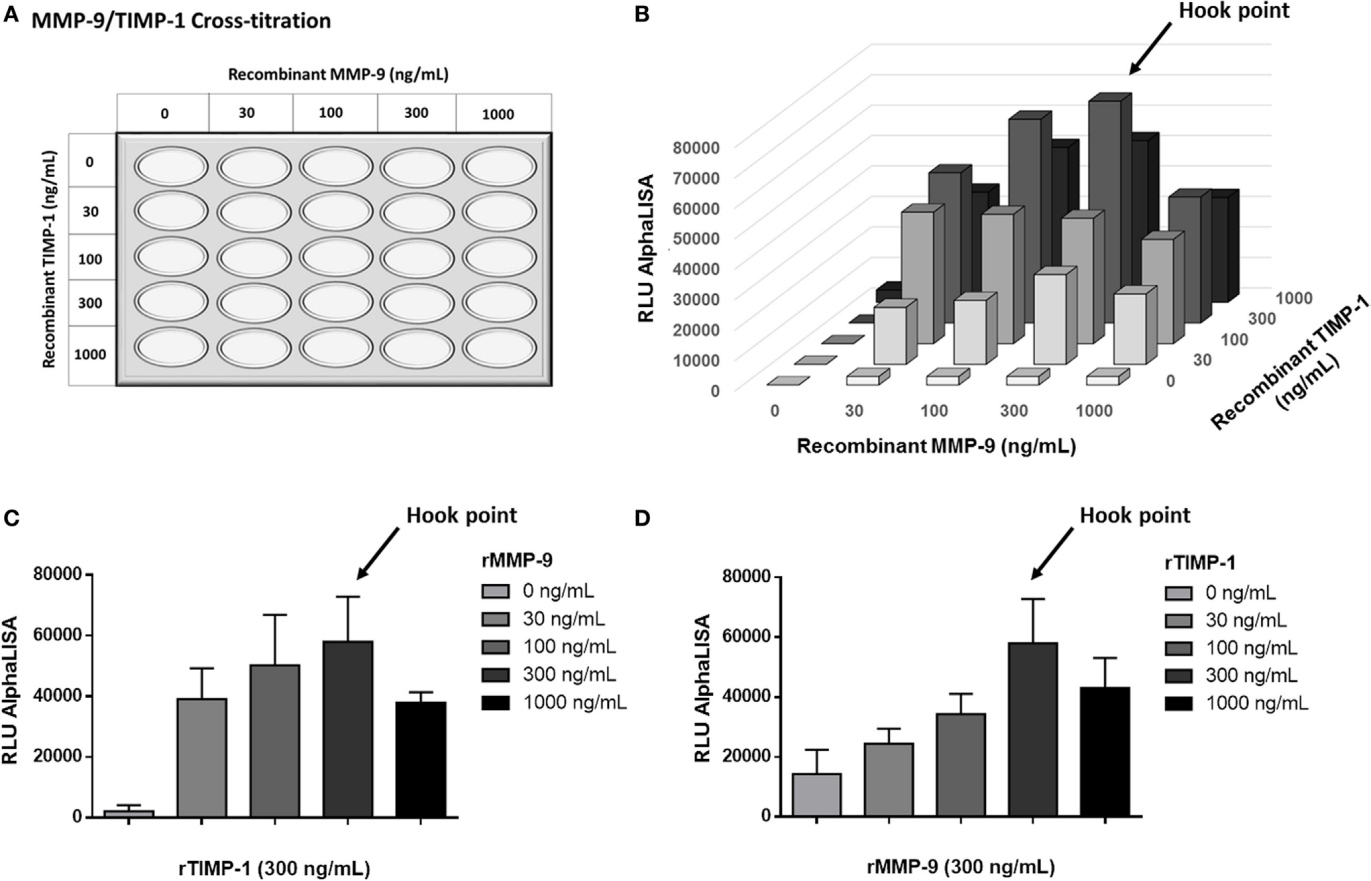
AlphaLISA^®^ matrix metalloproteinase (MMP)-9–tissue inhibitor of metalloproteinase (TIMP)-1 interaction immunoassay for detecting recombinant MMP-9 (rMMP-9) and TIMP-1 proteins. **(A)** Schematic illustration of the cross-titration MMP-9–TIMP-1 template. MMP-9 and TIMP-1 concentrations from 0 to 1,000 ng/mL were used for the optimization of the technique. **(B)** Results of the rMMP-9–TIMP-1 interaction immunoassay expressed as binding RLU (relative luminescence units) using different concentrations of MMP-9 and TIMP-1 (0, 30, 100, 300, and 1,000 ng/mL). **(C)** Curve 0–1,000 ng/mL of MMP-9 with a fixed dose of 300 ng/mL TIMP-1. **(D)** Curve 0–1,000 ng/mL of TIMP-1 with a fixed dose of 300 ng/mL MMP-9. **(C,D)** assays were repeated twice. Hook point is reached at 300 ng/mL MMP-9 and 300 ng/mL TIMP-1.

To optimize the AlphaLISA^®^ immunoassay to determine MMP-9–TIMP-1 complexes, first we tested the protocol by incubating beads with serial concentrations of recombinant human MMP-9 and TIMP-1 proteins (final concentration: 0, 30, 100, 300, and 1,000 ng/mL). The cross-titration scheme used is shown in Figure 4A, and the specific interactions observed are shown in Figure 4B. As endogenous MMP-9 and TIMP-1 bind in a 1:1 stoichiometry, measured luminescence (expressed as binding relative light units or RLU) was higher when MMP-9 and TIMP-1 were, at least, at the same concentration. In the absence of one of the proteins, the luminescence signal was 0 or very close to 0 after subtracting the blank. We observed that the optimal AlphaLISA^®^ signal for MMP-9 and TIMP-1 interaction corresponded to a concentration of rMMP-9 and TIMP-1 of 300 ng/mL, and excess protein above this concentration led to a decrease in the signal (Figures 4C,D). Thus, the named hook point was reached at 300 ng/mL MMP-9 and 300 ng/mL TIMP-1. At this hook point, both donor and acceptor beads are saturated and the maximum RLU signal is observed. As shown in Figures 4C,D, an excess of protein provokes an inhibition of the association between MMP-9 and TIMP-1, causing a decrease in RLU signal as shown at 1,000 ng/mL MMP-9 and 1,000 ng/mL TIMP-1. Finally, the hook effect is also observed when any of the two proteins MMP-9 and TIMP-1 was used in a concentration higher than the hook point (300 ng/mL), demonstrating that an excess of any protein involved in the interaction can oversaturate the donor and acceptor beads and reduce the interaction signal.

We believe that this AlphaLISA^®^ MMP-9–TIMP-1 immunoassay using recombinant proteins may be very useful in pharmacological competition assays especially in the field of basic research. With this type of AlphaLISA^®^ immunoassay, the interaction capacity of other potential MMP-9 inhibitors beyond TIMP-1 might be analyzed and compared to the interaction capacity exerted by the purified human rTIMP-1. For pharmacological competition assays, we recommend to use a concentration of MMP-9 and TIMP-1 below the hook point.

### AlphaLISA^®^ Immunoassay to Detect Endogenous/Native MMP-9 and TIMP-1 Interactions in Human Plasma

**Figure 5:**
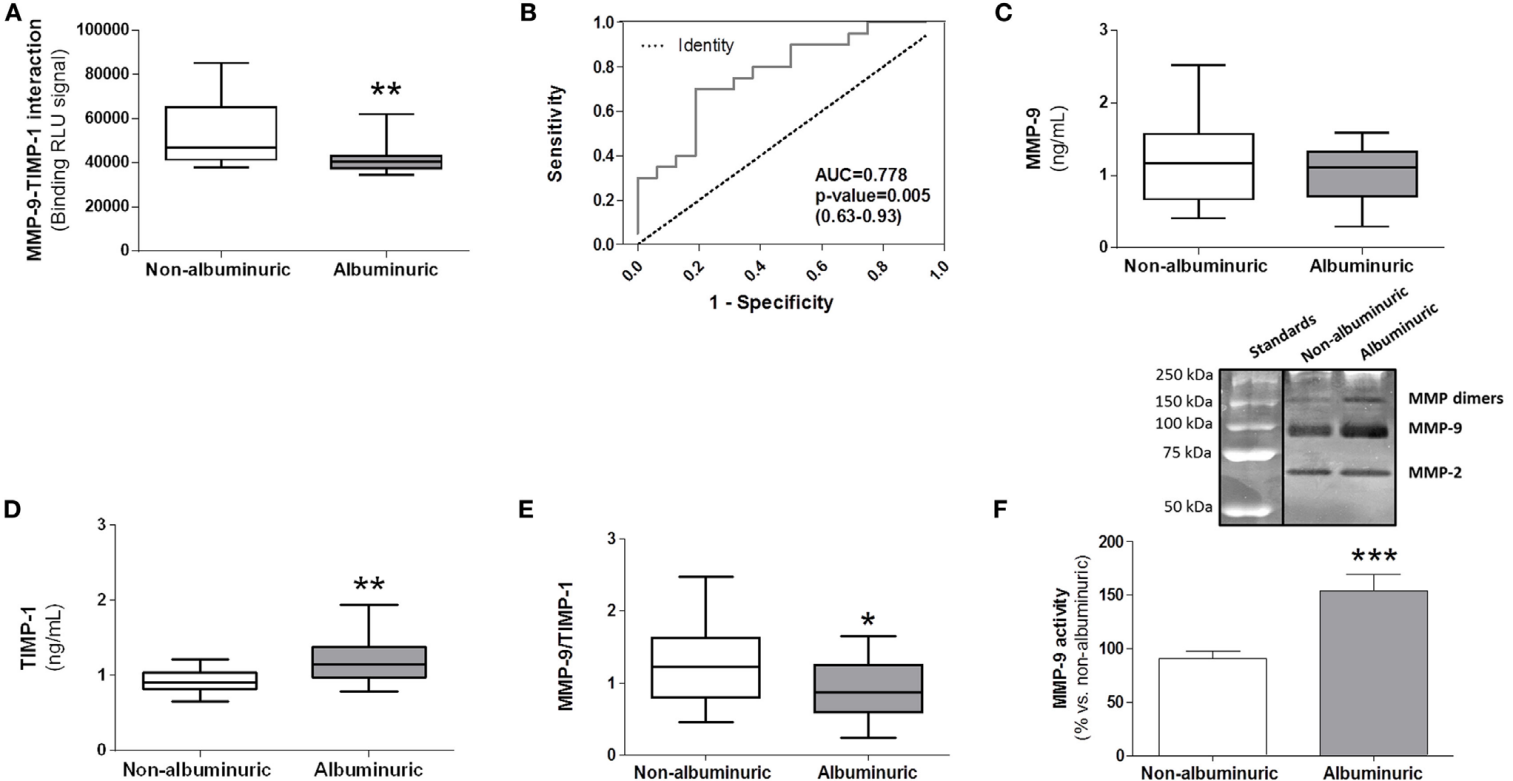
Comparison between specific matrix metalloproteinase (MMP)-9–tissue inhibitor of metalloproteinase (TIMP)-1 interactions determined by AlphaLISA^®^ immunoassay and MMP-9, TIMP-1, and MMP-9/TIMP-1 ratio levels estimated by classical ELISAs in human plasma samples from hypertensive patients with normoalbuminuria versus albuminuria. **(A)** AlphaLISA^®^ MMP-9–TIMP-1 interaction immunoassay expressed as binding RLU (relative luminescence units) in normoalbuminuric (*n* = 16) and albuminuric (*n* = 20) patients. **(B)** Receiver-operating characteristics curve for AlphaLISA^®^ MMP-9–TIMP-1 immunoassay (AUC = 0.778, 95% CI 0.63–0.93). This curve was used for comparing the ability of the AlphaLISA^®^ MMP-9–TIMP-1 immunoassay to predict albuminuria in hypertensive patients. AUC, area under curve; CI, confidence interval. **(C)** MMP-9 and **(D)** TIMP-1 levels quantified by standard ELISAs. **(E)** MMP-9/TIMP-1 ratio estimation. *P < 0.05, **P < 0.01, and ***P < 0.001 versus albuminuric patients. **(F)** Gelatinase MMP-9 activity in human plasma, upper panel, representative gelatin zymography gel, bottom panel, densitometric analysis expressed as % of change versus normoalbuminuria. In panels **(A,C,D,E)**, results are expressed as median values (horizontal line) and the interquartile ranges (box). In panel **(F)**, results are expressed in bar graph as mean values ± SEM.

The purpose of this new MMP-9–TIMP-1 AlphaLISA^®^ immuno assay is to allow the direct determination of native MMP-9–TIMP-1 complexes in circulating blood as biofluid in a quick, highly simplified, and cost-effective manner. Moreover, as TIMP-1 is involved in the regulation of MMP-9 activity, it is important to consider the interaction between MMP-9 and TIMP-1 when MMP-9 activity is altered in a specific pathology. To directly evaluate this, we used the AlphaLISA^®^ immunoassay to detect endogenous/native MMP-9–TIMP-1 complexes in human plasma from patients with different pathophysiological conditions in which MMP-9 activity was altered. Representative data from MMP-9–TIMP-1 AlphaLISA^®^ interactions in human plasma samples are shown in a study recently published by our laboratory (16). Similar to that study, here we analyzed MMP-9–TIMP-1 immunocomplexes in plasma samples from hypertensive patients with or without albuminuria as an indicator of endothelial dysfunction and arterial stiffness (22–25). We found that plasma from patients with albuminuria had significantly less MMP-9–TIMP-1 complexes than equivalent samples from non-albuminuric patients (Figure 5A; *P* < 0.01). We performed receiver-operating characteristics (ROC) analysis to assess the discriminatory capacity of the immunoassay to detect MMP-9–TMP-1 complexes between non-albuminuric hypertensive patients and those that develop albuminuria. The ROC area was 0.778 (Figure 5B; *P* = 0.005), indicating that the AlphaLISA^®^ immunoassay satisfactorily discriminates albuminuric from non-albuminuric patients.

We compared the results using the AlphaLISA^®^ MMP-9–TIMP-1 immunoassay with ELISAs as gold standard analytical methods for estimating MMP-9 and TIMP-1 in human plasma. Results showed that MMP-9 protein levels were similar between albuminuric and non-albuminuric patients (Figure 5C). Surprisingly, TIMP-1 protein levels were significantly higher in albuminuric than in non-albuminuric patients (*P* < 0.01) (Figure 5D), which resulted in a significantly lower MMP-9/TIMP-1 ratio (*P* < 0.05) (Figure 5E), and contradicted the results from the AlphaLISA^®^ immunoassay (Figure 5A). In agreement with these conflicting findings, other studies have demonstrated that MMP-9 activity shows no consistency with the calculated MMP-9/TIMP-1 ratio in the preclinical setting (14, 26). Therefore, results obtained using classical ELISAs for MMP-9 and TIMP-1 should not be translated to estimate MMP-9 activity through the use of MMP-9/TIMP-1 ratios. The use of the calculated MMP-9/TIMP-1 ratio is therefore not always a good surrogate measurement because it underestimates the actual MMP-9–TIMP-1 interaction and thus the true MMP-9 activity. As the interaction between the N-terminal domain of TIMP-1 and the active center site of MMP-9 inhibits MMP-9 proteolytic activity (27), the decreased interaction between the two detected in the plasma of albuminuric patients may result in increased MMP-9 proteolytic activity. To determine this, we carried out a zymography analysis of plasma to analyze MMP-9 gelatinase activity. A representative zymogram of plasma samples from the two patient groups is shown in Figure 5F, upper panel. Gelatinase MMP-9 activity was significantly higher in albuminuric than in non-albuminuric patients (*P* < 0.001), supporting the results obtained with AlphaLISA^®^ MMP-9–TIMP-1 immunoassay (Figure 5A). However, the activity of MMP-9 quantified by zymography should be viewed with important caution, because TIMP-1 is chemically separated from MMP-9 during the SDS-PAGE zymography process, not being possible to know how much TIMP-1 was endogenously bound to MMP-9 in plasma samples. This limitation is resolved in our new AlphaLISA^®^ immunoassay in which MMP-9–TIMP-1 complexes are directly detected. As mentioned, one possible explanation for the contradictory results obtained with the classical ELISAs (Figures 5C–E) compared to our AlphaLISA^®^ MMP-9–TIMP-1 immunoassay (Figure 5A) would be that an indirect estimation of MMP-9/TIMP-1 ratios does not consider that the biological function of TIMP-1 can be regulated by posttranslational modifications such as oxidation of the N-terminal domain (15). Indeed, we previously observed that the plasma level of oxidized TIMP-1 (denominated oxy-TIMP-1) is higher in albuminuric patients than in normoalbuminuria patients (16), which explains the significant decrease in MMP-9–TIMP-1 interactions observed in hypertensive patients when albuminuria is present (Figure 5A). In addition, it is important to point out that to analyze independently MMP-9 and TIMP-1 levels using commercial ELISAs, it is necessary to use more human plasma volume instead of 5 µL as required when we used this AlphaLISA^®^ protocol.

On the other hand, it is interesting to note that the AlphaLISA^®^ technology is highly versatile and useful in preclinical research, allowing the design of new immunoassay protocols to detect proteins (28–31) or interactions between proteins (32, 33) contained in human plasma samples. One example is its use to detect the interaction between hepatitis C virus-encoded NS5A and cyclophilin A as a basis for inhibitor screening (32). In the cardiovascular research area, an AlphaLISA^®^ immunoassay has been used to specifically detect N-terminal pro-B-type natriuretic peptide (NT-proBNP) in plasma of heart failure patients (31). In the clinical practice, there is no consensus about the circulating fragments of NT-proBNP derived from the precursor proBNP that are specifically secreted from the failing heart. This situation can lead to the misdiagnosis of heart failure. The authors employed different AlphaLISA^®^ immunoassays to improve the accuracy of the different circulating forms on NT-proBNP in plasma from these patients. As in these studies, here we have described in detail the proven protocol with step-by-step procedures of an automated, simple, and fast immunoassay previously used in our laboratory (16) to measure MMP-9–TIMP-1 complexes using AlphaLISA^®^ technology.

### Limitations

One limitation of assays based in chemiluminescence-dependent responses might be the presence of prooxidant and antioxidant components contained in serum/plasma samples, which could alter the luminescence signal (34). Therefore, these components might be potential pitfalls of assays based in luminescence response. However, it has already been demonstrated by several authors that AlphaLISA^®^ assays are unaffected by interfering with antioxidant components as ascorbic acid or bilirubin as well as triglycerides contained in plasma samples (29, 35). In addition, here we have also tested that prooxidant components as H_2_O_2_ did not alter the level of RLU observed for the MMP-9–TIMP-1 interactions using rMMP-9 and TIMP-1 proteins (Figure S1A in Supplementary Material) or endogenous MMP-9–TIMP-1 interactions in human plasma samples (Figure S1B in Supplementary Material). Finally, the AlphaLISA^®^ immunoassay to measure endogenous MMP-9–TIMP-1 complexes does not reveal a quantitative interaction since it is a semi-quantitative technique. For this reason, the use of control samples as reference is absolutely necessary in order to determine whether there is more or less proportion of endogenous MMP-9–TIMP-1 interaction in a specific experimental or pathological condition.

In summary, our protocol increases throughput and laboratory productivity by considerably decreasing hands-on assay time. In addition, as the protocol directly measures MMP-9–TIMP-1 interactions, there is no need to estimate the MMP-9/TIMP-1 ratio, which may be unreliable and could underestimate the actual MMP-9 activity in pathological circumstances, as illustrated here in conditions of albuminuria. We believe that this new protocol will facilitate the immunodetection of these biomarkers and will have a high potential for use in basic, preclinical research.

## Ethics Statement

In our study, a total of 36 patients aged >18 years with primary hypertension were recruited from the Hypertension Unit at Hospital Universitario 12 de Octubre, Madrid. Exclusion criteria included patients with diabetes mellitus or primary hyperaldosteronism. Patients were classified as normoalbuminuria when albumin/creatinine ratio was maintained <20 mg/g for men and <30 mg/g for women, and as albuminuria for those patients who reported abnormal urinary albumin excretion that remained above target levels (albumin/creatinine ratio ≥20 mg/g for men and ≥30 mg/g for women). The study was approved by the Ethics Committee of the Hospital Universitario 12 de Octubre and was conducted according to the principles of the Declaration of Helsinki. All patients signed a written informed consent before inclusion.

## Author Contributions

Conceived and designed the experiments: GR-H. Performed experiments and analyzed the data: HP-O, ER-S, JN-G, and GR-H. Design of study in patients and selection of patients: MB, GA-L, and JS. Contributed reagents/materials: MF-V, LR, and GR-H. Wrote the manuscript: HP-O, LR, and GR-H. Collaborated in elaborating the last version of the manuscript: ER-S and JN-G. New experiments and the necessary work to answer all questions requested by the reviewers: ER-S, JN-G and GR-H.

## Conflict of Interest Statement

The authors declare that the research was conducted in the absence of any commercial or financial relationships that could be construed as a potential conflict of interest.
