# Supplementary material for: Rapid, Automated, and Specific Immunoassay to Directly Measure Matrix Metalloproteinase-9–Tissue Inhibitor of Metalloproteinase-1 Interactions in Human Plasma Using AlphaLISA Technology: A New Alternative to Classical ELISA
Source: Front Immunol. 2017 Jul 24;8:853. doi: 10.3389/fimmu.2017.00853 (PMC5523156; doi:10.3389/fimmu.2017.00853)
Supplement: Supplementary file 1 [file image_1.pdf]

METHODS

Zymography

Blood samples were centrifuged at 900 g for 10 min at 4ºC to obtain plasma and stored at -80ºC until assays. Total protein concentration was measured with Bradford method (BioRad). Gels were stained with Coomassie blue (BioRad) and then destained. Gelatinolytic activity of MMP-9 was detected as transparent bands against the background of the blue-stained gels. Transparent bands are the result of the digestion of gelatin, the substrate incorporated in polyacrylamide gel, as a result of enzymatic activity of MMP-9.

Quantification of MMP-9 and TIMP-1 proteins levels by ELISA

Plasma concentration levels of MMP-9 and TIMP-1 were measured using commercial ELISA tests specific for each proteins: Quantikine® ELISA Human MMP-9 Immunoassay (catalog number: DPM900 from R&D Systems) and Quantikine® ELISA Human TIMP-1 Immunoassay (catalog number: DTM100 from R&D Systems). These assays were carried out following manufacturer's instructions.

RESULTS

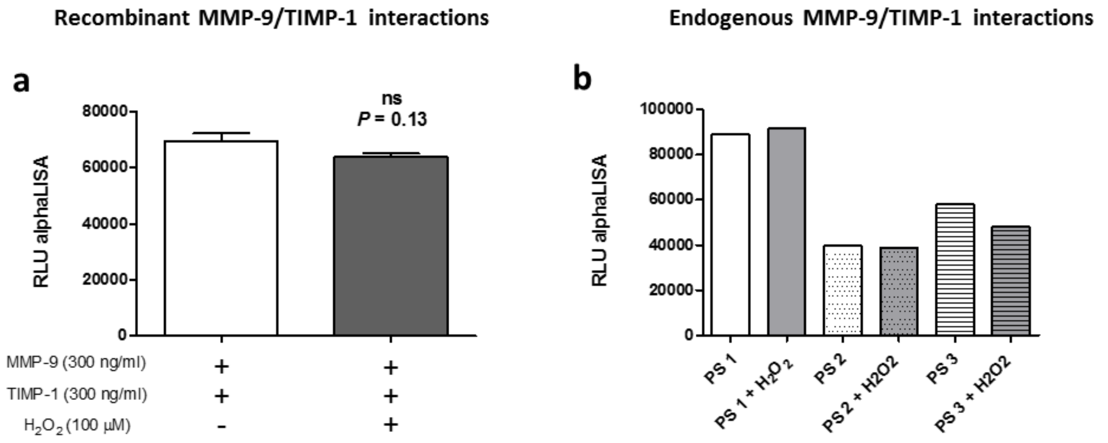

Figure 1. Pro-oxidant molecules as H_2O_2 do not interfere with the RLU signal in this AlphaLISA assay to measure purified recombinant or endogenous MMP-9-TIMP-1 interactions. (a) Assay with recombinant MMP-9 and TIMP-1 proteins or **(b)** with endogenous MMP-9 and TIMP-1 proteins contained in human plasma samples. In both assays samples were pre-incubated with 100 µM H_2O_2 during 30 min. For recombinant proteins we replicated the assay three times. For endogenous proteins we carried out the assay in three independent human plasma samples. PS=plasma sample. Ns=not significant.
